# The long noncoding RNA *LAL* contributes to salinity tolerance by modulating *LHCB1s’* expression in *Medicago truncatula*

**DOI:** 10.1038/s42003-024-05953-9

**Published:** 2024-03-08

**Authors:** Yang Zhao, Yafei Liu, Feiran Zhang, Zeng-Yu Wang, Kirankumar S. Mysore, Jiangqi Wen, Chuanen Zhou

**Affiliations:** 1 The Key Laboratory of Plant Development and Environmental Adaptation Biology, Ministry of Education, School of Life Science, Shandong University, Qingdao, P.R. China; 2 Grassland Agri-Husbandry Research Center, College of Grassland Science, Qingdao Agricultural University, Qingdao, 266109 China; 3 Institute of Agricultural Biosciences, Oklahoma State University, 3210 Sam Noble Parkway, Ardmore, OK 73401 USA

**Keywords:** Gene regulation, Abiotic

## Abstract

Long non-coding RNAs (lncRNAs) are abundant in plants, however, their regulatory roles remain unclear in most biological processes, such as response in salinity stress which is harm to plant production. Here we show a lncRNA in *Medicago truncatula* identified from salt-treated Medicago *truncatula* is important for salinity tolerance. We name the lncRNA *LAL*, *LncRNA* *ANTISENSE* to *M. truncatula* *LIGHT-HARVESTING CHLOROPHYLL A/B BINDING* (*MtLHCB*) *genes. LAL* is an antisense to four consecutive *MtLHCB* genes on chromosome 6. In salt-treated *M. truncatula*, *LAL* is suppressed in an early stage but induced later; this pattern is opposite to that of the four *MtLHCB*s. The *lal* mutants show enhanced salinity tolerance, while overexpressing *LAL* disrupts this superior tolerance in the *lal* background, which indicates its regulatory role in salinity response. The regulatory role of *LAL* on *MtLHCB1.4* is further verified by transient co-expression of *LAL* and *MtLHCB1.4-GFP* in tobacco leaves, in which the cleavage of *MtLHCB1.4* and production of secondary interfering RNA is identified. This work demonstrates a lncRNA, *LAL*, functioning as a regulator that fine-tunes salinity tolerance via regulating *MtLHCB1s*’ expression in *M. truncatula*.

## Abstract (web summary)

A study on long non-coding RNAs (lncRNAs) shows a lncRNA in *Medicago truncatula* is important for salinity tolerance. Mutants for the lncRNA show enhanced salinity tolerance through up-regulating light-harvesting chlorophyll a/b binding (*MtLHCB*) genes.

## Introduction

Long noncoding RNA (lncRNA) are transcripts longer than 200 nucleotides that contain small reading frames or encode no peptides^1^. They are generated mostly by RNA polymerase II, while some by RNA polymerase I and RNA polymerase III^2^. According to their positions relative to coding genes, lncRNAs can be categorized into several classes: antisense of the coding genes, intronic, intergenic, upstream of the coding gene’s promoter, promoter-associated and transcription start site-associated lncRNAs, et al^3^. Recent studies have demonstrated their roles in transcriptional and post-transcriptional regulations^4^. Transcriptional regulation is very important for plants, because plants cannot move to avoid harm from the environment. Recently, more and more lncRNAs have been found to play roles in receiving signals from the outside and responding to different stresses, through proteins modifications and endo-siRNAs generation^5^. Therefore, lncRNAs play important roles, yet the one in salinity stress remains unclear.

In the past two decades, numerous studies have been conducted on plant salinity response, especially in the model plant *Arabidopsis thaliana*, and uncovered a number of key players involved in stress signaling and response^6^. In *A. thaliana*, the imposition of abiotic stresses leads to a transient burst of excess reactive oxygen species (ROS) production, and the disruption to ROS homeostasis has a negative effect on stress tolerance and on plant’s growth^7,8^. On the first hand, the genes encoding the plant plasma membrane-localized nicotinamide adenine dinucleotide phosphate reduced form (NADPH) oxidases, *RESPIRATORY BURST OXIDASE HOMOLOGUE*s (*RBOH*s), are involved in ROS production when plants are imposed to stresses^9,10^. Production and accumulation of ROS, such as superoxide (O_2_^-^) and hydrogen peroxide (H_2_O_2_), cause oxidative damages in cells, such as apoplastic compartments and cellular membranes. The damages caused by ROS can be measured by the content of malondialdehyde (MDA). Secondly, ROS can function as signaling molecules to activate the expression of downstream salinity stress-responsive genes. On the second hand, both ABA synthetic and signaling pathways are activated when a plant is exposed to salinity stress^6,8^. In Arabidopsis, *ABA1* encodes a zeathanxin epoxidase to catalyze zeathanxin to violaxanthin. Then violaxanthin is catalyzed into neoxanthin by neoxanthin synthase and neoxanthin to xanthoxin by 9-CIS-EPOXYCAROTENOID DIOXYGENASE (NCED).

In Arabidopsis, the chloroplast is important for ROS production^11^. In chloroplasts, photosystem II (PSII) outer antenna proteins consist of LHCBs. Among them, LHCB1, LHCB2 and LHCB3, form the major antenna complexes^12^. Electrons are produced from PSII and passed to photosystem I (PSI). Until now, the role of *LHCB1*s in salinity tolerance has not been elucidated clearly, neither the regulators of *LHCB1*s in salt stress.

Here in this work, we identified a lncRNA, *LncRNA as an* *ANTISENSE for MtLHCB1s* (*LAL*), from salt-treated Medicago plants, which was an antisense for four *MtLHCB1* genes in a tandem. The Medicago *lal* mutants that disrupted the lncRNA’s expression showed enhanced salinity tolerance accompanied with elevated *MtLHCB1*s’ expression and activated ROS and ABA pathways. The transient expression assays of *LAL* with *MtLHCB1.4* and knockdown of *MtLHCB1s*’ plants diminishing *lal*’s salinity tolerance, which showed that *MtLHCB1s* were targets of *LAL*. These findings demonstrate the important regulatory role of *LAL* in Medicago via modulate *MtLHCB1*s’ expression under salinity tolerance.

## Results

### Upregulation of *MtLHCB1*s from salt-treated *M. truncatula*

**Fig. 1:**
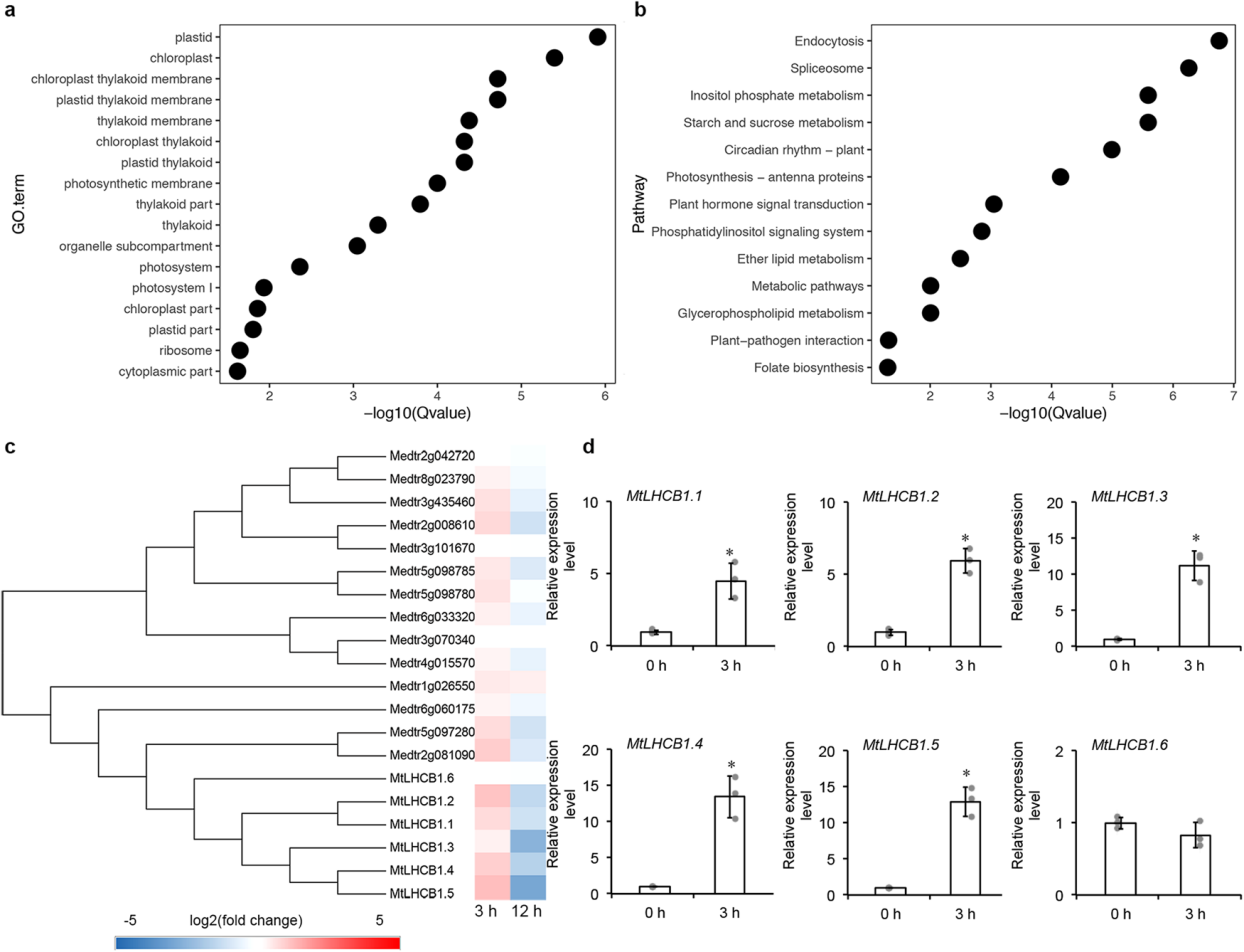
Enrichment analysis of *M. truncatula* WT plants after NaCl treatment. **a** The GO enrichment analysis of DEGs at three hours after NaCl treatment. **b** The KEGG enrichment analysis of DEGs at three hours after NaCl treatment. **c** The heatmap of MtLHCBs and the homologs at three hours and 12 hours after NaCl treatment. The qRT validation of *MtLHCB1.1*, *MtLHCB1.2*, *MtLHCB1.3*, *MtLHCB1.4*, *MtLHCB1.5* and *MtLHCB1.6* (**d**) at three hours after NaCl treatment. *MtUBIQUITIN* was used as the internal control. The expression level at 0 hour was set at 1.0. Error bars represent the SD from three biological replicates (grey dots). Columns labeled with asterisks indicate significant differences from those in WT (*P < 0.05, student’s *t* test). *n* = 3.

To study the mechanism underlying *M. truncatula*’s response to salinity, we carried out a transcriptomic analysis of NaCl-treated plants. By comparing the mock- and NaCl-treated plants, we identified 4,412 differentially expressed genes (DEGs) with over two-fold changes in expression and false discovery rates (FDR) smaller than 0.001, including 1026 upregulated and 1814 downregulated DEGs at three hours post-treatment, and 1021 upregulated and 551 downregulated at 12 hours (Supplementary Data. 1). Gene Ontology (GO) enrichment analysis revealed that genes encoding chloroplast-localized proteins were significantly enriched in the DEGs (*q* value < 0.01, Fig. 1a). Kyoto Encyclopedia of Genes and Genomes (KEGG) enrichment analysis revealed the similar pattern with the photosynthesis pathway significantly enriched in the DEGs (*q* value < 0.01, Fig. 1b). The most differentially regulated DEGs included 18 *MtLHCBs* involved in photosynthesis regulated at three hours and 12 hours with similar trends (Fig. 1c).

Phylogenetic analysis of LHCBs from *M. truncatula* and *A. thaliana* grouped 18 MtLHCBs into seven subgroups (Supplementary Fig. 1), in which *MtLHCB1s* showed the most induction in their expression. Among the five *MtLHCB1s* in the *M. truncatula* genome, *MtLHCB1.1*, *MtLHCB1.2*, *MtLHCB1.3*, *MtLHCB1.4*, and *MtLHCB1.5* showed a similar induction in their expressions after NaCl treatment, and the former four genes are located on chromosome 6 as a tandem array (Fig. 1d). This result suggested the involvement *MtLHCB1s* in salinity tolerance in Medicago.

### A long noncoding RNA, *LAL*, was identified antisense for *MtLHCB1*s

**Fig. 2:**
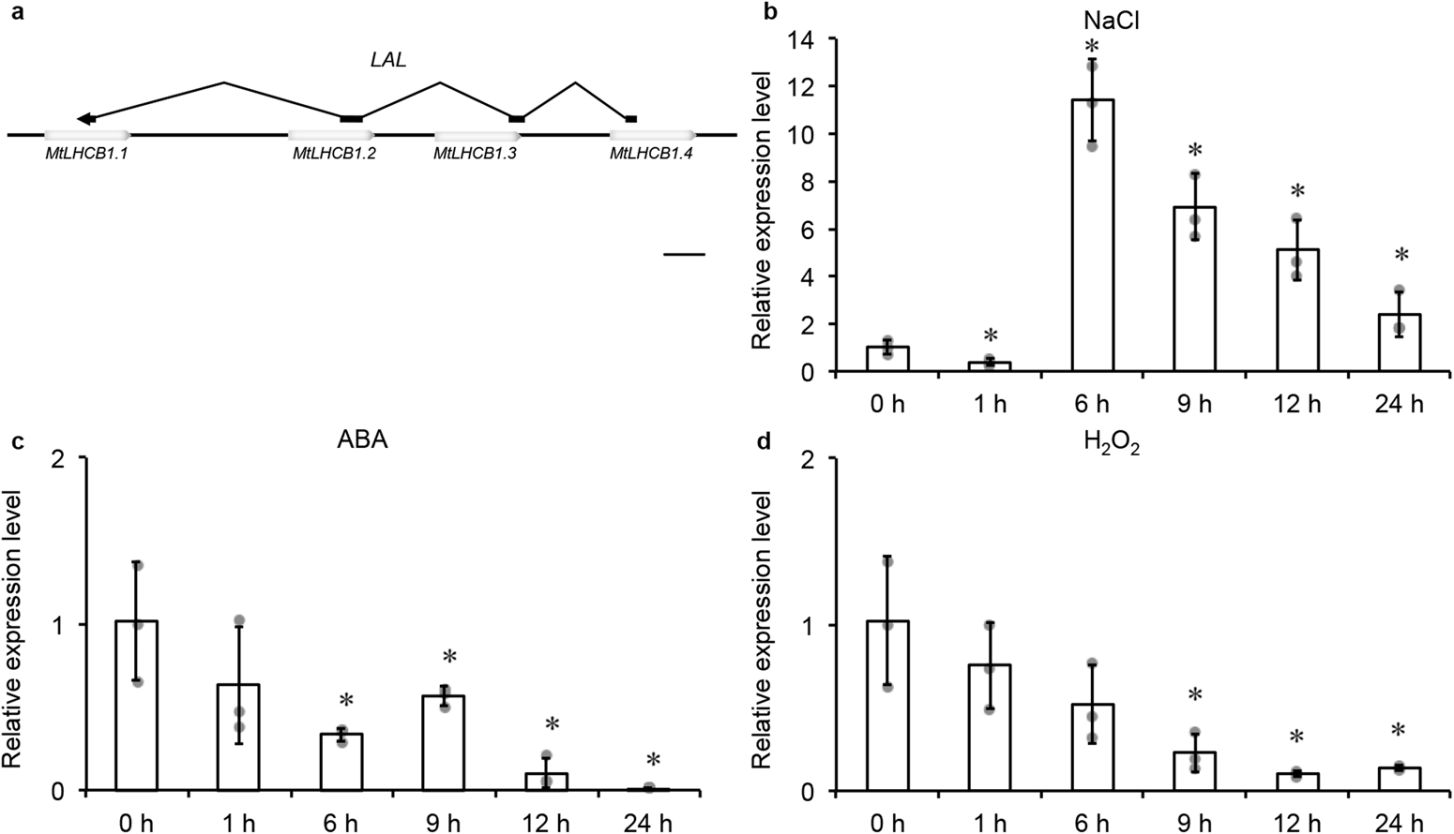
*LAL*’*s* expression under salinity, ABA and H_2_O_2_ treatment. **a** The scheme of *LAL* and *MtLHCB1.1*, *MtLHCB1.2*, *MtLHCB1.3* and *MtLHCB1.4* genes structure. Empty boxes represent *MtLHCB1*s, and black boxes represent *LAL*. Bar = 400 bp. **b** The expression level of *LAL* at different time points by qRT-PCR in *M. truncatula* WT plants under salinity treatment. **c** The expression level of *LAL* at different time points by qRT-PCR in *M. truncatula* WT plants under ABA treatment. **d** The expression level of *LAL* at different time points by qRT-PCR in *M. truncatula* WT plants under H_2_O_2_ treatment. *MtUBIQUITIN* was used as the internal control. The expression level in 0 hour was set at 1.0. Error bars represent the SD from three biological replicates (grey dots). Columns labeled with asterisks indicate significant differences from those in 0 hour (*P < 0.05, student’s *t* test). *n* = 3.

From the alignment of RNA sequencing data, we noticed an antisense transcript overlapped with *MtLHCB1* genes on chromosome 6 (Supplementary Fig. 2). We cloned the full length of this transcript and verified its ends using 5’- and 3’-RACE. Sanger sequencing revealed a 446-bp transcript consists of four exons, which was predicted to encode no protein by Coding Potential Calculator (CPC). Because the four exons of *LAL* were antisense for the tandem array of four *MtLHCB1* genes, we named this gene as *LONG NON-CODING RNA AS AN ANTISENSE FOR MtLHCB1s* (*LAL*) (Fig. 2a).

Expression assay revealed that *LAL* displayed a ubiquitous expression pattern with higher transcription levels in leaf. The four *MtLHCB1*s were mainly expressed in leaf tissue, while *MtLHCB1.5* was mostly expressed in flower, pod and root (Supplementary Fig. 3). We therefore focused on *MtLHCB1.1-1.4* on chromosome 6.

In salinity-treated plants, *LAL* expression was suppressed at three hours but then activated more than ten times at six hours, followed by a gradual reduction to the basal level at 24 hours (Fig. 2b). In contrast, the *MtLHCB1.1-1.4* exhibited an opposite pattern with more than ten-fold induction at three hours after salinity treatment and experienced a slow decline till 24 hours (Supplementary Fig. 4). As both *LAL* and *MtLHCB1*s responded to salinity treatment, we examined their response to ABA and H_2_O_2_. The result showed that the expression of *LAL* was suppressed and *MtLHCB1*s transcriptional levels induced under ABA or H_2_O_2_ treatment (Fig. 2c, d and Supplementary Fig. 4). Our results identified a non-coding RNA, *LAL*, responding to salinities stress signals during salinity, ABA and H_2_O_2_ treatment.

### Disruption of *LAL* conferred enhanced salinity tolerance in *lal-1*

**Fig. 3:**
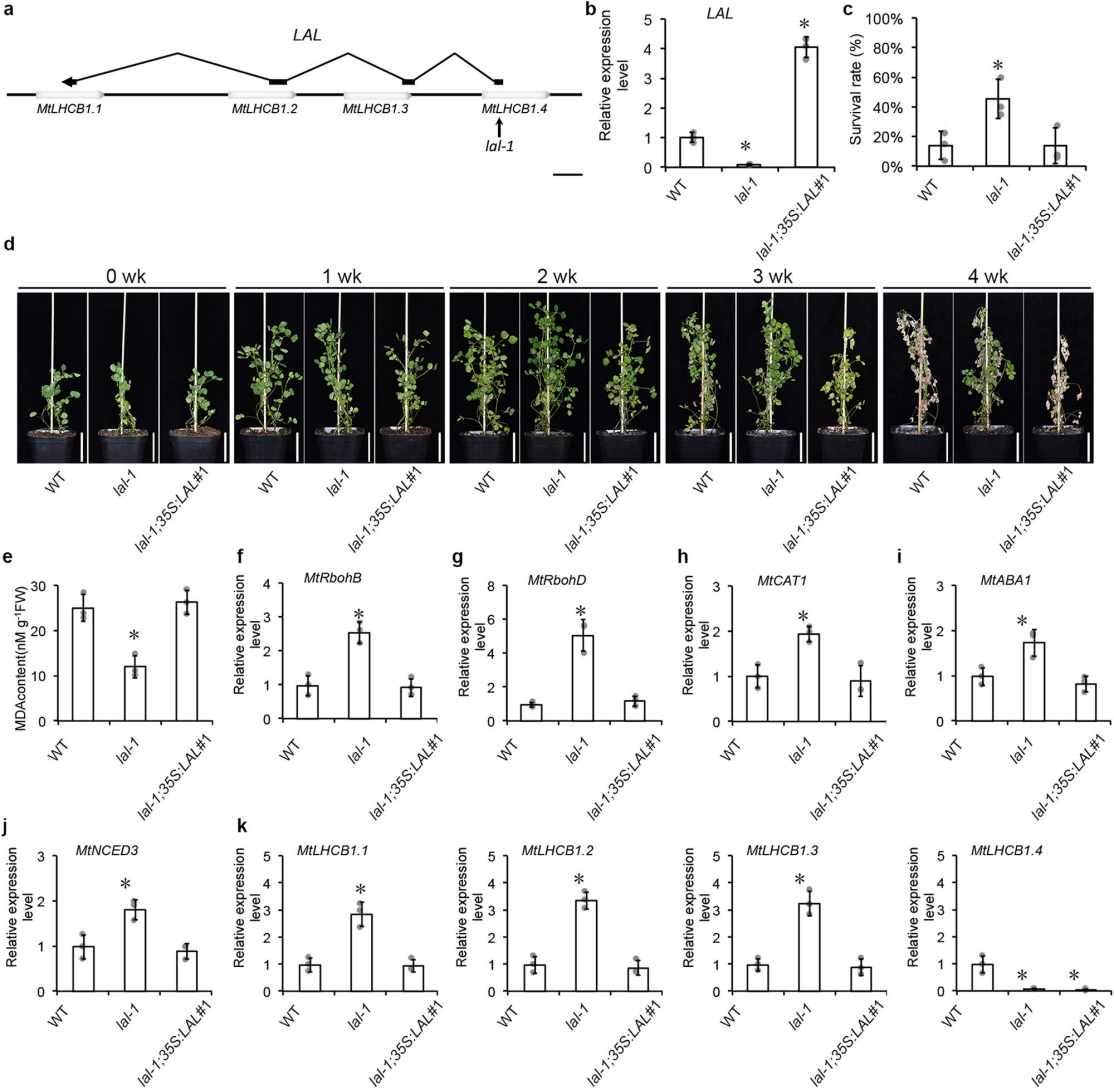
Overexpression *LAL* disrupted the superior salinity tolerance in *lal-1*. **a** The scheme of *LAL* and *MtLHCB1.1*, *MtLHCB1.2*, *MtLHCB1.3* and *MtLHCB1.4* genes structure and *Tnt1* insertion in the gene of *lal-1*. Empty boxes represent *MtLHCB1*s, and black boxes represent *LAL*. One arrow indicates the position where *Tnt1* inserts in *LAL*. Bar = 400 bp. **b** The expression level of *LAL* in WT, *lal-1* and *lal-1;35* *S:LAL*#1 by qRT-PCR. **c** The plant survival rates measured after the four-week treatment of salinity. Error bars represent the SD from three biological replicates (grey dots). Columns labeled with asterisks indicate significant differences from those in WT (*P < 0.05, student’s *t* test). **d** Four-week-old plants of WT, *lal-1* and *lal-1;35* *S:LAL*#1 plants after a four-week successive exposure to 50, 100, 150 and 200 mM NaCl. Bar = 5 cm. **e** The MDA content of WT, WT, *lal-1* and *lal-1;35* *S:LAL*#1. The expression level of *MtRbohB* (**f**), *MtRbohD* (**g**), *MtCAT1* (h), *MtABA1* (**i**) and *MtNCED3* (**j**) in WT, *lal-1* and *lal-1;35* *S:LAL*#1 by qRT-PCR. The expression level of *MtLHCB1.1*, *MtLHCB1.2*, *MtLHCB1.3*, and *MtLHCB1.4* (**k**) in WT, *lal-1* and *lal-1;35* *S:LAL*#1 by qRT-PCR. *MtUBIQUITIN* was used as the internal control. The expression level in WT was set at 1.0. Error bars represent the SD from three biological replicates (grey dots). Columns labeled with asterisks indicate significant differences from those in WT (*P < 0.05, student’s *t* test). *n* = 3.

To study the role of *LAL* in salinity response, we screened *M. truncatula Tnt1* mutant collection and found *lal-1*. *lal-1* had an insertion in the *LAL*’s first exon and also the *MtLHCB1.4*’s coding sequence, which disrupted both genes’ functions (Fig. 3a, b). To assess the salinity tolerance in *lal-1*, we conducted a consecutive 4-week NaCl treatment and found that the mutant was tolerant to salinity with a high survival rate and stayed green till the end of the treatment (Fig. 3c, d). The indicator of oxidative damage, MDA, were assessed in *lal-1* and wild-type (WT) plants, and the result showed a significantly lower contents of MDA in *lal-1* compared with WT (Fig. 3e). The expressions of *MtRboh* genes involved the ROS synthesis pathway, including *MtRbohB* (*Medtr3g098380*), *MtRbohD* (*Medtr3g098320*) and a ROS scavenger gene, *MtCAT1* (*Medtr3g115370*) were activated in *lal-1* (Fig. 3f–h). As ABA plays a crucial role in salinity response, we further assessed the expression of genes involved in ABA synthesis. The genes in the ABA synthesis pathway, *MtABA1* (*Medtr5g017350*), and *MtNCED3* (*Medtr2g070460*) were significantly higher in *lal-1* than those in the WT plants (Fig. 3i, j).

To verify the superior salinity tolerance in *lal-1* was caused by its mutation, we overexpressed the *LAL* transcript in the homozygous *lal-1* background. The *lal-1;35* *S:LAL*#1, *lal-1;35* *S:LAL*#2 and *lal-1;35* *S:LAL*#3 transgenic lines restored the expression of *LAL* (Fig. 3b and Supplementary Fig. 5), with suppression of *MtLHCB1.1*, *MtLHCB1.2* and *MtLHCB1.3* compared with *lal-1* but undetectable expression in *MtLHCB1.4* (Fig. 3k–n). To assess the salinity tolerance in the transgenic plants, we conducted the same consecutive four-week NaCl treatment and found that the *lal-1;35* *S:LAL* transgenic plants showed chlorosis and growth retardment like WT. The average survival rates of *lal-1;35* *S:LAL*#1 and WT plants was 13% and 14%, respectively, which was significantly lower than 44% in *lal-1* that stayed green by the end of NaCl treatment (Fig. 3c). MDA were assessed with comparable contents in WT and *lal-1;35* *S:LAL*#1 (Fig. 3e). These results demonstrated that knockout of *LAL* conferred a superior tolerance to salt stress and overexpressing *LAL* diminished superior salinity tolerance in *lal-1*.

*lal-1* showed enhanced salinity tolerance and had an insertion located both in *LAL* and *MtLHCB1.4*. To learn the role of *MtLHCB1.4* in salinity tolerance, we identified a *mtlhcb1.4* mutant that carried one *Tnt1* insertion in the CDS of *MtLHCB1.4* located 179 bp before the transcriptional start site of *LAL*. *mtlhcb1.4* possessed an insertion in *MtLHCB1.4*, but not in *LAL* (Supplementary Fig. 6a). The expression result showed that the expression of *MtLHCB1.4* was completely disrupted in *mtlhcb1.4*. We overexpressed *MtLHCB1.4* in the WT background and the resulting transgenic plants were tested the expression of *MtLHCB1.4* (Supplementary Fig. 6b). The four-week-old WT, *mtlhcb1.4* and transgenic plants were treated with a saline gradient NaCl to test their salinity tolerance. The mutant showed comparable salinity survival rate. Meanwhile, the overexpressing *MtLHCB1.4* plants *35* *S:MtLHCB1.4*#1 and #2 measured showed enhanced salinity tolerance (Supplementary Fig. 6c). The expressions of *MtRboh* genes and ROS scavenging *MtCAT1* were elevated and the enzyme activity of MtCAT1 increased in transgenic plants (Supplementary Fig. 6d–g). The expressions of *MtABA1* and *MtNCED3* in ABA synthesis were increased in transgenic plants (Supplementary Fig. 6h, j). These results demonstrated the positive role of *MtLHCB1.4* in salinity tolerance.

### *lal* mutants displayed salinity tolerance with synthesis of ROS and ABA enhanced

**Fig. 4:**
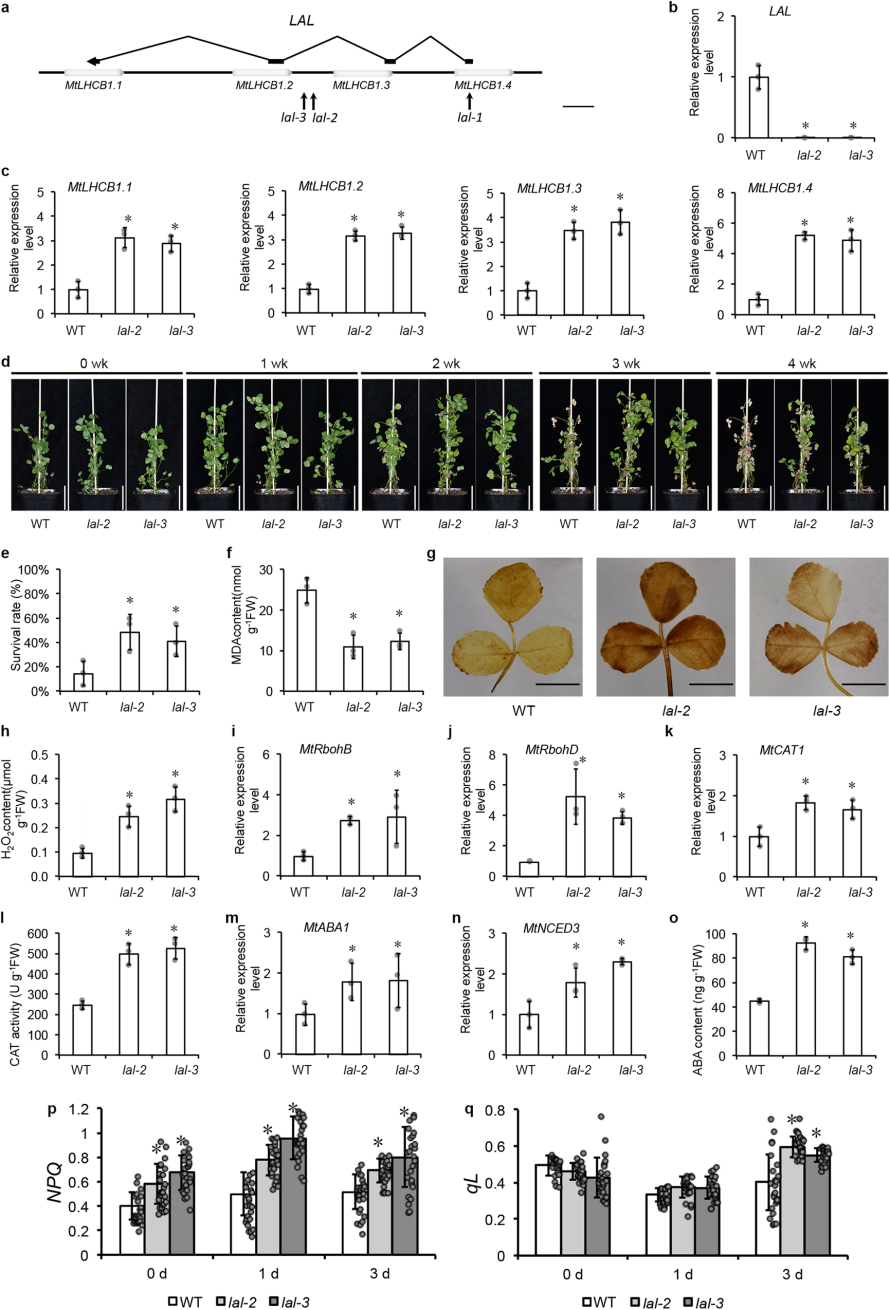
*lal* showed salinity tolerance with enhanced ROS and ABA synthesis pathways. **a** The scheme of *LAL* and *MtLHCB1.1*, *MtLHCB1.2*, *MtLHCB1.3* and *MtLHCB1.4* genes structure and *Tnt1* insertions in *LAL* of *lal-1*, *lal-2* and *lal-3*. Empty boxes represent *MtLHCB1*s, and black boxes represent *LAL*. Three arrows indicate the positions where *Tnt1* inserts in *LAL*. Bar = 400 bp. The expression level of *LAL* (**b**) and *MtLHCB1s* (**c**) in *lal-2* and *lal-3* by qRT-PCR. **d** Four-week-old plants of WT, *lal-2* and *lal-3* mutants after a four-week successive exposure to 50, 100, 150 and 200 mM NaCl. Bar = 5 cm. **e** The plant survival rates of WT, *lal-2* and *lal-3* measured after the four-week treatment of salinity. **f** The MDA content of WT, *lal-2* and *lal-3* mutants. **g** The DAB staining of WT, *lal-2* and *lal-3* mutants. Bar = 1 cm. **h** The H_2_O_2_ contents of WT, *lal-2* and *lal-3* mutants. The expression level of *MtRbohB* (**i**), *MtRbohD* (**j**) and *MtCAT1* (**k**) in WT, *lal-2* and *lal-3* by qRT-PCR. **l** The CAT activity in WT, *lal-2* and *lal-3*. **m** The expression level of *MtABA1* in WT, *lal-2* and *lal-3* by qRT-PCR. **n** The expression level of *MtNCED3* in WT, *lal-2* and *lal-3* by qRT-PCR. **o** The ABA contents of WT, *lal-2* and *lal-3* mutants. Error bars represent the SD from three biological replicates (grey dots). Columns labeled with asterisks indicate significant differences from those in WT (*P < 0.05, student’s *t* test). *n* = 3. *MtUBIQUITIN* was used as the internal control in qRT-PCR. The expression level in WT was set at 1.0. Response to salinity stress on photosynthetic parameters *NPQ* (**p**) and *qL* (**q**) of the WT, *lal-2* and *lal-3* mutants at different time points of NaCl-treatment. Values are shown by mean ± SD (grey dots with black circles). Columns labeled with asterisks indicate significant differences from those in WT (*P < 0.05, student’s *t* test). *n* = 25.

To study the molecular function of *LAL* in salinity response, we screened our mutant collection and found another two mutants with *Tnt1* insertions within the *LAL* gene, *lal-1*, *lal-2* and *lal-3*. Both *lal-2* and *lal-3* had an insertion in the second intron of *LAL*, which corresponded to the intergenic region between *MtLHCB1.2* and *MtLHCB1.3* (Fig. 4a). In the homozygous *lal-2* and *lal-3* mutants, the *LAL* expression was undetectable while the expression levels of *MtLHCB1s* were significantly higher than those in WT, suggesting a negative regulation of *MtLHCBs* by *LAL* (Fig. 4b, c).

Salinity tolerance was assessed in the *lal-2* and *lal-3* mutants. The four-week-old plants were treated with a saline gradient with 50 to 200 mM NaCl for four weeks. The WT plants showed severe chlorosis at three week post-treatment, while the *lal* mutants stayed green. At the end the experiment, the WT plants showed severe chlorosis and growth retardment, but the most mutant plants remained green. The average survival rates of *lal-2* and *lal-3* after the NaCl treatment were 49% and 41%, respectively, which were significantly higher than that of 15% in WT (Fig. 4d, e). Moreover, the MDA contents, the indicator of oxidative damage, in the *lal* mutants were significantly lower than that in WT (Fig. 4f).

To understand the mechanism underlying salinity tolerance, we measured several hallmark responses to salt stress in the *lal* mutants. DAB staining detected higher ROS contents in *lal-2* and *lal-3* than that in the WT plants (Fig. 4g), accompanied with the elevated H_2_O_2_ contents (Fig. 4h) and the expressions of *MtRboh* genes involved the ROS pathway, including *MtRbohB*, *MtRbohD*, (Fig. 4i, j). The expression of *MtCAT1*, was significantly higher in *lal-2* and *lal-3* than WT (Fig. 4k), and its activity also showed a two-fold increase in the mutant leaves (Fig. 4l). These results showed that the ROS synthesis and scavenging pathways were constitutively activated in the *lal* mutants. *MtABA1*, and *MtNCED3* were significantly higher in *lal-2* and *lal-3* mutants than those in the WT plants (Fig. 4m, n). We also measured the ABA contents, and the result showed that ABA accumulated to higher levels in *lal* mutants compared to the WT plants (Fig. 4o). These results demonstrated ABA biosynthesis pathway was activated in *lal* mutants.

For MtLHCB1s are PSII outer antenna proteins, we investigated the impact of *LAL* on the photosynthetic apparatus by chlorophyll fluorescence parameters such as the maximal quantum yield of photosystem II (*Fv/Fm*), the photochemical yields of photosystem II (*YII*), non-photochemical quenching (*NPQ*) and photochemical quenching (*qL*). The four-week-old WT, *lal-2* and *lal-3* mutant plants were treated with 100 mM NaCl for three days. The decrease of *Fv/Fm* was because of the impaired photochemical activity in salt treatment. The WT and mutant plants showed comparable values of *Fv/Fm* (Supplementary Fig. 7a). *YII* reflected the impairment to photosystem II caused by salinity showing the similar trends with *Fv/Fm* in WT and mutant plants (Supplementary Fig. 7b). Interestingly, *NPQ*, transferring photons as heat to protect light-harvesting complexes, showed a remarkable increase in *lal-2* and *lal-3* mutants (Fig. 4p). In photosynthesis, the efficiency of light energy transferred to photochemistry, *qL* was remaining the high values in *lal-2* and *lal-3* (Fig. 4q). Hence, the photosynthesis of *lal* showed enhanced photoprotection under salinity stress.

### *LAL* performed salinity tolerance via regulating *MtLHCB1s*’ expression

**Fig. 5:**
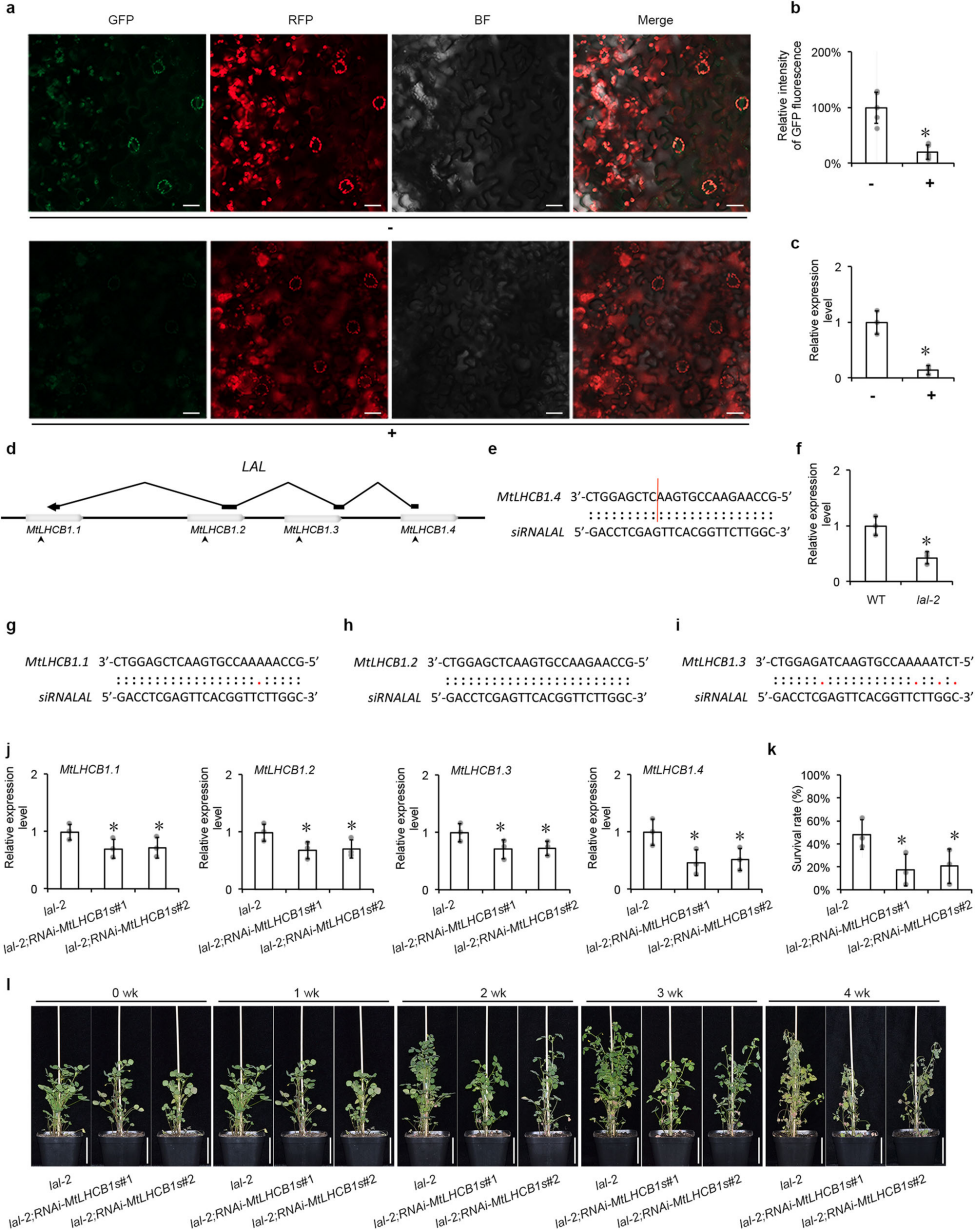
*LAL* regulated *MtLHCB1s* in salinity tolerance. **a** Transient expression assays of MtLHCB1.4 with *LAL* indicated by ‘*+*’ and without *LAL* indicated by ‘-’ in *N. benthamiana*. Bar = 20 µm. **b** The relative GFP fluorescence intensity of *35* *S:MtLHCB1.4-GFP* with and without *LAL*. Values are shown by mean ± SD (grey dots). *n* = 5. Columns labeled with asterisks indicate significant differences from those in *35* *S:MtLHCB1.4-GFP* without *LAL* (*P < 0.05, student’s *t* test). The intensity in *35* *S:MtLHCB1.4-GFP* without *LAL* was set at 100%. **c** The expression level of *MtLHCB1.4* with and without *LAL* by qRT-PCR. *L25* was used as the internal control. Error bars represent the SD from three biological replicates (grey dots). Columns labeled with asterisks indicate significant differences from those in *35* *S:MtLHCB1.4-GFP* without *LAL* (*P < 0.05, student’s *t* test). The expression level in *35* *S:MtLHCB1.4-GFP* without *LAL* was set at 1.0. **d** The scheme of *LAL* and *MtLHCB1.1*, *MtLHCB1.2*, *MtLHCB1.3* and *MtLHCB1.4* genes structure and the complementary sequences to *siRNALAL* in *MtLHCB1s*. Empty boxes represent *MtLHCB1*s, and black boxes represent *LAL*. Four arrowheads indicate the positions of complementary sequences to *siRNALAL*. **e** Complementary sequence of *MtLHCB1.4* and *siRNALAL*. Red vertical line indicates the cleavage site for *siRNALAL* validated by RLM 5’-RACE. **f** The expression level of *siRNALAL* in *lal-2* by qRT-PCR. *MtU6* was used as the internal control. The expression level in WT was set at 1.0. Error bars represent the SD from three biological replicates (grey dots). Columns labeled with asterisks indicate significant differences from those in WT (*P < 0.05, student’s *t* test). Complementary sequences of *MtLHCB1.1* (**g**), *MtLHCB1.2* (**h**) and *MtLHCB1.3* (**i**) to *siRNALAL*. **j** The expression level of *MtLHCB1.1*, *MtLHCB1.2*, *MtLHCB1.3* and *MtLHCB1.4* in *lal-2*, *lal-2;RNAi-MtLHCB1s*#1 and *lal-2;RNAi-MtLHCB1s*#2 plants by qRT-PCR. *MtUBIQUITIN* was used as the internal control. The expression level in *lal-2* was set at 1.0. Error bars represent the SD from three biological replicates (grey dots). Columns labeled with asterisks indicate significant differences from those in *lal-2* (*P < 0.05, student’s *t* test). **k** The plant survival rates measured after the four-week treatment of salinity. Error bars represent the SD from three biological replicates (grey dots). Columns labeled with asterisks indicate significant differences from those in *lal-2* (*P < 0.05, student’s *t* test). **l** Four-week-old plants of *lal-2*, *lal-2; RNAi-MtLHCB1s* #1 and *lal-2;RNAi-MtLHCB1s*#2 plants after a four-week *s*uccessive exposure to 50, 100, 150 and 200 mM NaCl. Bar = 5 cm. *n* = 3.

To further study the relationship between *LAL* and *MtLHCB1*, the transient expression assay of *LAL* and *MtLHCB1.4-GFP* was investigated in tobacco leaves (Fig. 5a). The results showed the GFP signal intensity of MtLHCB1.4 transferred with *LAL* was diminished in transfection with *35* *S:LAL* compared with that without *LAL* (Fig. 5b). The suppression of *MtLHCB1.4-GFP* expression was verified by qRT-PCR (Fig. 5c and Supplementary Fig. 8). These results indicate a suppression role of *LAL* on *MtLHCB1.4*.

To further determine if *LAL* cause the post-transcriptional regulation of *MtLHCB1s*, we carried out 5’-RACE in the tobacco leaves co-expressing *MtLHCB1.4* and *LAL* and found the cleavage products of the *MtLHCB1.4*. In the tobacco leaves co-expressing *MtLHCB1.4* and *LAL*, we detected a siRNA, *siRNALAL* corresponding to the *MtLHCB1.4* cleavage site (Fig. 5e). The abundance of *siRNALAL* was also found in a lesser extent in *lal-2* compared with WT (Fig. 5f). Due to the high identities among these MtLHCB1s, we constructed *MtLHCB1s*-RNAi lines in *lal-2* backgrounds and a total of sixteen transgenic lines were generated. The qRT-PCR results showed that *MtLHCB1.1*, *MtLHCB1.2*, *MtLHCB1.3* and *MtLHCB1.4* were statistical significantly suppressed (Fig. 5j). The salinity tolerance was assessed in these RNAi lines along with *lal-2*. The results showed the salinity tolerance observed in *lal-2* were diminished in *lal-2;RNAi-MtLHCB1s*#1 and *lal-2;RNAi-MtLHCB1s*#2 with the survival rates about 18% and 21%, statistical significantly lower than that of *lal-2*, about 48% (Fig. 5k). Hence, the salinity tolerance of *lal* mutant relied on *MtLHCB1s* functions.

## Discussion

Here we identified a lncRNA in *M. truncatula*, *LAL*, an antisense to four consecutive *MtLHCB* genes on chromosome 6. *lal-2 and lal-3* mutants showed enhanced salinity tolerance accompanied with elevated *MtLHCB1*s’ expression and activated ROS and ABA pathways. The knockdown of *MtLHCB1s*’ plants diminished *lal*’s salinity tolerance, showing that *MtLHCB1s* were targets of *LAL*.

*LHCB1s* encode apoproteins of photosystem II and are important for plants. But, the role of LHCB1 in salinity tolerance was unclear. It is reported previously that in Arabidopsis *lhcb1* was insensitive to ABA^13,14^, and ABA activated the expressions of *LHCBs* in low concentration, and suppressed their expressions in high concentration, which accumulated in the process of salinity stress. It was shown in our study that overexpressing *MtLHCB1.4* enhanced salinity tolerance. In overexpressing *MtLHCB1.4* plants, the ROS synthesis and scavenging pathways were both activated, while the biosynthesis pathway of ABA was enhanced (Supplementary Fig. 6). Our findings help to understand the positive roles of MtLHCB1s protein in plant salinity tolerance and ROS homeostasis.

In Arabidopsis, LHCB1 has 5 homologs, LHCB1.1, LHCB1.2, LHCB1.3, LHCB1.4 and LHCB1.5, among which three are located on chromosome 1, two on chromosome 2^15^. Take MtLHCB1.4, for example, MtLHCB1.4 is the homolog of LHCB1s, with identities higher than 80% and the homolog of MtLHCB1s, with identities higher than 93%. Interestingly, salinity-responsive *MtLHCB1s* form a cluster showing a tandem pattern in genome. The tandem-located targets with high identities were also found in *MtCBFs* in cold tolerance^16^. In our study, four *MtLHCB1s* shared similar expression pattern (Supplementary Fig. 3) and similar stress responses to *LAL* (Fig. 2 and Supplementary Fig. 4). Hence *LAL* is the antisense of these tandem-located *MtLHCB1s*, this *LAL-MtLHCB1s* module makes multiple *MtLHCB1s* salinity-responsive simultaneously.

In this work, when *MtLHCB1.4* was co-expressed with *LAL*, the signals of MtLHCB1.4 were diminished, which suggested the sense-antisense regulation between *LAL* and *MtLHCB1.4* (Fig. 5). In mammals, every cell of female embryo in early developmental stages only keeps one functional X chromosome from the two from parents and silences the other one^17^. The lncRNAs *Xist* initiates the silencing and the antisense *Tsix* blocks it on the active X. These two partners, *Xist* and *Tsix*, form complementary duplexes processed by Dicer into small RNAs^18^. In our work, *siRNALAL* complemented to *MtLHCB1s* and its expression was diminished in *lal-2* mutant (Fig. 5). The disruption of complementation between *LAL* and *MtLHCB1s* probably led to decrease of siRNAs and upregulation of *MtLHCB1s*. Interestingly, in *lal-1;35* *S:LAL*#1, the expressions of *MtLHCB1.1*, *MtLHCB1.2*, and *MtLHCB1.3* were reduced and the salinity tolerance vanished (Fig. 3). Via the cluster-assembling targeting *MtLHCB1s* homologs, *LAL* performed salinity tolerance in a dosage-depending manner. This finding helps to guide agricultural engineering in alfafa.

## Methods

### Plant materials and growth conditions

The plant materials used in this work were in the *M. truncatula* ecotype R108 background. Mutants were requested from the Noble Research Institute *Tnt1* Mutant collection^19^. The *lal-1*/NF20242, *lal-2*/NF19927, *lal-3*/NF17218, and *mtlhcb1.4*/NF14097 mutants were identified using gene-specific and *Tnt1*-specific primers (listed in Supplementary Table 1). Seeds were scarified mechanically and vernalized at 4 °C on moist filter paper for one week before transfer to soil. Plants were grown in a growth chamber at 22 °C under a 16-h-light/8-h-dark cycle with 150 μmol/m^2^/s light intensity and 70-80% relative humidity. To determine gene expression in different tissues, root, stem, leaf, flower and shoot tip were harvested from four-week-old plants. Pods were harvested from eight-week-old plants. For each biological replicate, 25 plants were used. Three biological replicates were included.

### Salinity treatment

For stress treatments, four-week-old plants were treated with 100 mM NaCl, 100 µM ABA or 1 mM H_2_O_2_. Leaf samples were harvested at different time points after treatment for RNA extraction. Salinity tolerance was scored after a four-week NaCl treatment starting with one-week treatment with 50 mM NaCl and an increment of 50 mM per week till 200 mM^20^. For each biological replicate, 25 plants were used. Three biological replicates were included.

### Phylogenetic analysis

To identify the homologs of MtLHCB1.4, we use the amino acid sequences of MtLHCB1.4 for a BLASTP search in *A. thaliana* and *M. truncatula* (http:// blast.ncbi.nlm.nih.gov/Blast.cgi). Eighteen homologous sequences, Medtr6g011870/MtLHCB1.1, Medtr6g011880/MtLHCB1.2, Medtr6g011890/MtLHCB1.3, Medtr6g012110/MtLHCB1.5, Medtr4g094605/MtLHCB1.6, Medtr2g008610, Medtr3g101670, Medtr2g042720, Medtr8g023790, Medtr3g070340, Medtr4g015570, Medtr3g435460, Medtr5g098785, Medtr5g097280, Medtr2g081090, Medtr1g026550, Medtr6g033320, and Medtr6g060175 were obtained from Medicago, and 21 homologous sequences, At1g29910/LHCB1.2, At1g29920/LHCB1.1, At1g29930/LHCB1.3, At2g34420/LHCB1.5, At2g34430/LHCB1.4, At2g05070/LHCB2.2, At2g05100/LHCB2.1, At3g27690/LHCB2.3, At1g15820/LHCB6, At1g76570/LHCB7, At2g40100/LHCB4.3, At3g08940/LHCB4.2, At4g10340/LHCB5, At5g01530/LHCB4.1, At5g54270/LHCB3, At1g19150/LHCA6, At1g45474/LHCA5, At1g61520/LHCA3, At3g47470/LHCA4, At3g54890/LHCA1, and At3g61470/LHCA2 from Arabidopsis. To study the phylogenetic relationships, the protein sequences were aligned in the ClustalW program (https://www.genome.jp/tools-bin/clustalw), and a neighbor-joining phylogenic tree was constructed with 1,000 bootstrap replicates in MEGA6.

### RNA isolation and quantitative real-time PCR (qRT-PCR)

Harvested tissue samples were frozen immediately in liquid nitrogen, from which RNA was extracted using RNeasy Mini Kit (Qiagen, U.S.A.) and treated with DNase I. RNA quantity was measured on a NanoDrop 2000 Spectrophotometer (NanoDrop Technologies, U.S.A). One µg of total RNA was transcribed into cDNA using ThermoScript RT-PCR system (ThermoFisher, U.S.A), and qRT-PCR was performed on a CFX Connect Real-Time PCR Detection system (Bio-Rad, U.S.A) using SYBR Green PCR Master Mix (Roche, Switzerland) in three biological replicates. A *MtUBIQUITIN* gene, *Medtr3g110110*, and a tobacco gene *L25*, Genbank:L18908.1, were used as internal controls^21^. siRNA cDNA was transcribed using miRcute Plus miRNA First-Strand cDNA kit (TIANGEN, China), and qRT-PCR was performed using miRcute Plus miRNA qPCR kit (TIANGEN, China) in three biological replicates, with 25 plants in each replicate. And *MtU6* gene, was used as an internal control^22^. Relative gene expression was calculated according to the *∆∆C*^*T*^ method. The primer sequences used for qRT-PCR were designed using the Primer Express 3.0 software and listed in Supplementary Table 1.

### Transcriptomic analysis

For the transcriptomic analysis, four-week-old plants were treated with water or 100-mM NaCl, and the leaf samples were harvested at three hours and 12 hours after treatment from mock- and NaCl-treated plants. Three biological replicates were included, with 25 plants in each replicate. RNA was extracted from the leaf samples as described above. RNA libraries were constructed and sequenced on a BGISEQ-500 platform following the manufacturer’s instructions (BGI Genomics, China). Raw reads were filtered by SOAPnuke (v1.5.2; https://github.com/BGI-flexlab/SOAPnuke), and then aligned to the *M. truncatula* reference transcriptome (version 4.0) by Bowtie2 (v2.2.5) using the default parameters. Read counts were calculated using RSEM^23^, and differentially expressed genes (fold change >= 2 and false discovery rate (FDR) < 0.001) were called using the R package DEGseq. Enrichment of Gene Ontology (GO) terms and KEGG pathways were assessed using a hypergeometric test and the *p* values were adjusted by the FDR method^24–26^. CPC2 was used for the CPC analysis^27^.

### Plasmid construction and plant transformation

The full length of *LAL*(Genbank accession number OR463063) transcript was confirmed by 5’ and 3’ rapid-amplification of cDNA ends (RACE) using SMARTer RACE 5’/3’ kit (Takara, Japan)^28^. The gene-specific primers (listed in Supplementary Table 1) were designed based on the transcript sequence and used to clone *LAL* into a pENTR-TOPO vector. The resulting pENTR-*LAL* plasmid was recombined with a destination vector pEarleyGate100 or pCAMBIA3301 to produce construct *35* *S:LAL*. The coding sequence (CDS) of *MtLHCB1.4* was amplified from a cDNA sample prepared from leaf RNA with c-*MtLHCB1.4*-forward and reverse primers and then used to generate pENTR*-MtLHCB1.4*, *35* *S:MtLHCB1.4* and *35* *S:MtLHCB1.4-GFP* constructs (listed in Supplementary Table 1) with destination vectors pEarleyGate100 and pEarleyGate103^29^. A 220-bp exon of *MtLHCB1.4* was cloned into pH7GW1WG2 to generate the *35* *S:MtLHCB1s*-RNAi construct.

For *M. truncatula* transformation, target constructs were introduced into an Agrobacterium strain EHA105 and then used to transform Medicago leaf disc^30^. The transgenic plants were genotyped with a 35 S primer and a gene-specific primer (listed in Supplementary Table 1). The positive transformants were accessed for the target gene expression by qRT-PCR.

### Transient expression assays

*35* *S:LAL* and *35* *S:MtLHCB1.4-GFP* were used for transient transformation of *Nicotiana benthamiana*^17^. Briefly, four-week-old *N. benthamiana* plants were infiltrated with *Agrobacterium* EHA105 containing *35* *S:MtLHCB1.4-GFP* (at an OD_600_ value of 0.4) of volume 1 mL per plant or 1 mL *35* *S: MtLHCB1.4-GFP* (at an OD_600_ value of 0.4) with 1 mL *35* *S:LAL* (at an OD_600_ value of 0.4) per plant and incubated in the dark at 25 °C for 24 hours. Leaves with comparable *BAR* expression were observed at 488-nm wavelength for the GFP fluorescence using a Zeiss LSM900 confocal microscopy (Zeiss, Germany). At least five images were taken under identical conditions and the relative intensity of GFP was assessed using the ImageJ software. The RNA of leaves co-expressing *LAL* and *MtLHCB1.4* were extracted and the RLM 5’-RACE (RNA ligase mediated rapid amplification of 5’ cDNA end) were performed with FirstChoice RLM-RACE kit (Invitrogen, U.S.A)^22^. Three biological replicates were included, with 25 plants in each replicate.

### Statistics and reproducibility

Data processing and statistical analysis were performed using Microsoft Excel 2010. A student’s *t*-test was used to evaluate the significance of the differences between two samples^31^. Three biological replicates were included, with 25 plants in each replicate.

### Determination of peroxide level, catalase activity and ABA content

H_2_O_2_ levels were visualized by DAB staining of leaf samples from four-week-old plants. H_2_O_2_ levels and catalase activities were measured with commercial kits purchased from Beyotime Institute of Biotechnology (Haimen, China). ABA contents of leaf samples from four-week-old plants were assessed by LC-MS-MS^7^. Three biological replicates were included, with 25 plants in each replicate.

### Chlorophyll fluorescence measurements

Measurements were carried out using a PAM-2500 (Heinz Walz GmbH, Germany) pulse amplitude modulation fluorometer. The plants were dark-adapted before the measurements and photosynthetic parameters were calculated by the PamWin software with provided automated induction and light curve routine^15^. Three biological replicates were included, with 25 plants in each replicate.

### Reporting summary

Further information on research design is available in the Nature Portfolio Reporting Summary linked to this article.

### Supplementary information

Supplementary File

Description of Additional Supplementary Files

Supplementary Data 1

Supplementary Data 2

Reporting Summary

Supplementary Table 1

## Data Availability

Data sets generated during this study are available at SRA with BioSample accession number PRJNA1067001. All other data are available from the corresponding author on reasonable request. Numerical source data for Figures is available in Supplementary Data 2.
