# Supplementary material for: The long noncoding RNA LAL contributes to salinity tolerance by modulating LHCB1s’ expression in Medicago truncatula
Source: Commun Biol. 2024 Mar 8;7:289. doi: 10.1038/s42003-024-05953-9 (PMC10923924; doi:10.1038/s42003-024-05953-9)
Supplement: Supplementary file 1 — Supplementary File [file 42003_2024_5953_MOESM1_ESM.pdf]

The long noncoding RNA *LAL* contributes to salinity tolerance by modulating *LHCB1s'* expression in *Medicago truncatula*

Yang Zhao[1], Yafei Liu[1], Feiran Zhang[1], Zeng-Yu Wang[2], Kirankumar S. Mysore[3], Jiangqi Wen[3] and Chuanen Zhou[1,*]

[1] The Key Laboratory of Plant Development and Environmental Adaptation Biology, Ministry of Education, School of Life Science, Shandong University, Qingdao, P.R. China

[2] Grassland Agri-Husbandry Research Center, College of Grassland Science, Qingdao Agricultural University, Qingdao 266109, China

[3] Institute of Agricultural Biosciences, Oklahoma State University, 3210 Sam Noble Parkway, Ardmore, Oklahoma 73401, USA

[*]corresponding author:czhou@sdu.edu.cn

Supplementary information

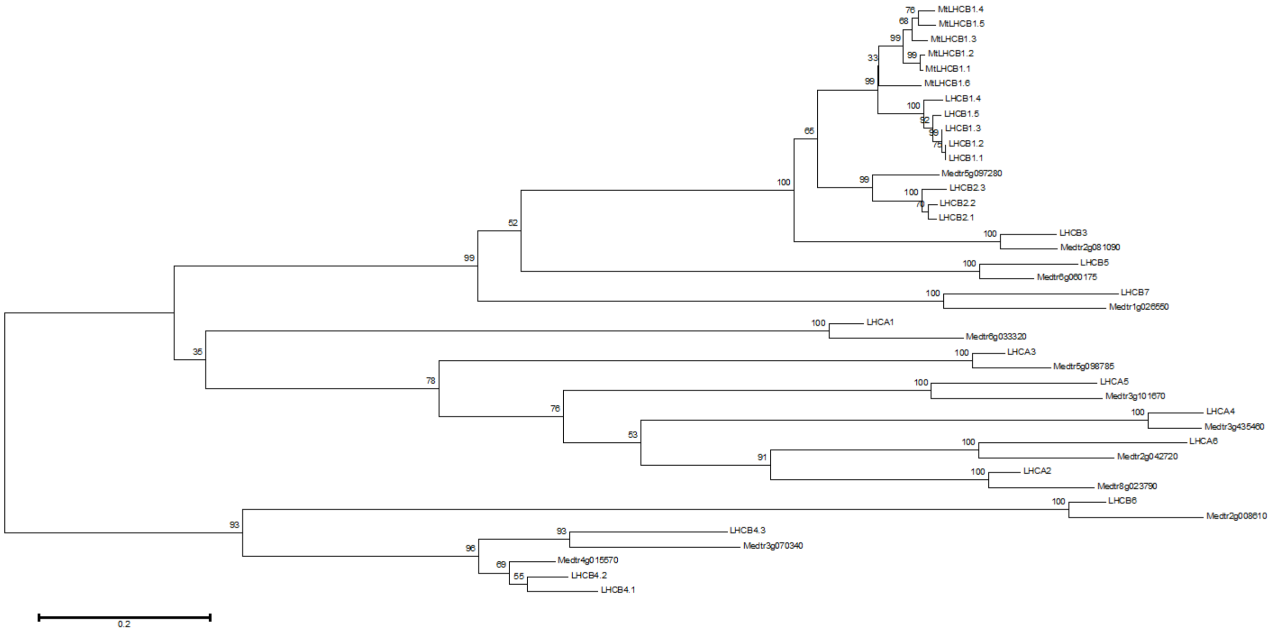

Supplementary Fig. 1 The phylogenetic tree of MtLHCB1s and the homologs in *M. truncatula* and *A. thaliana.*

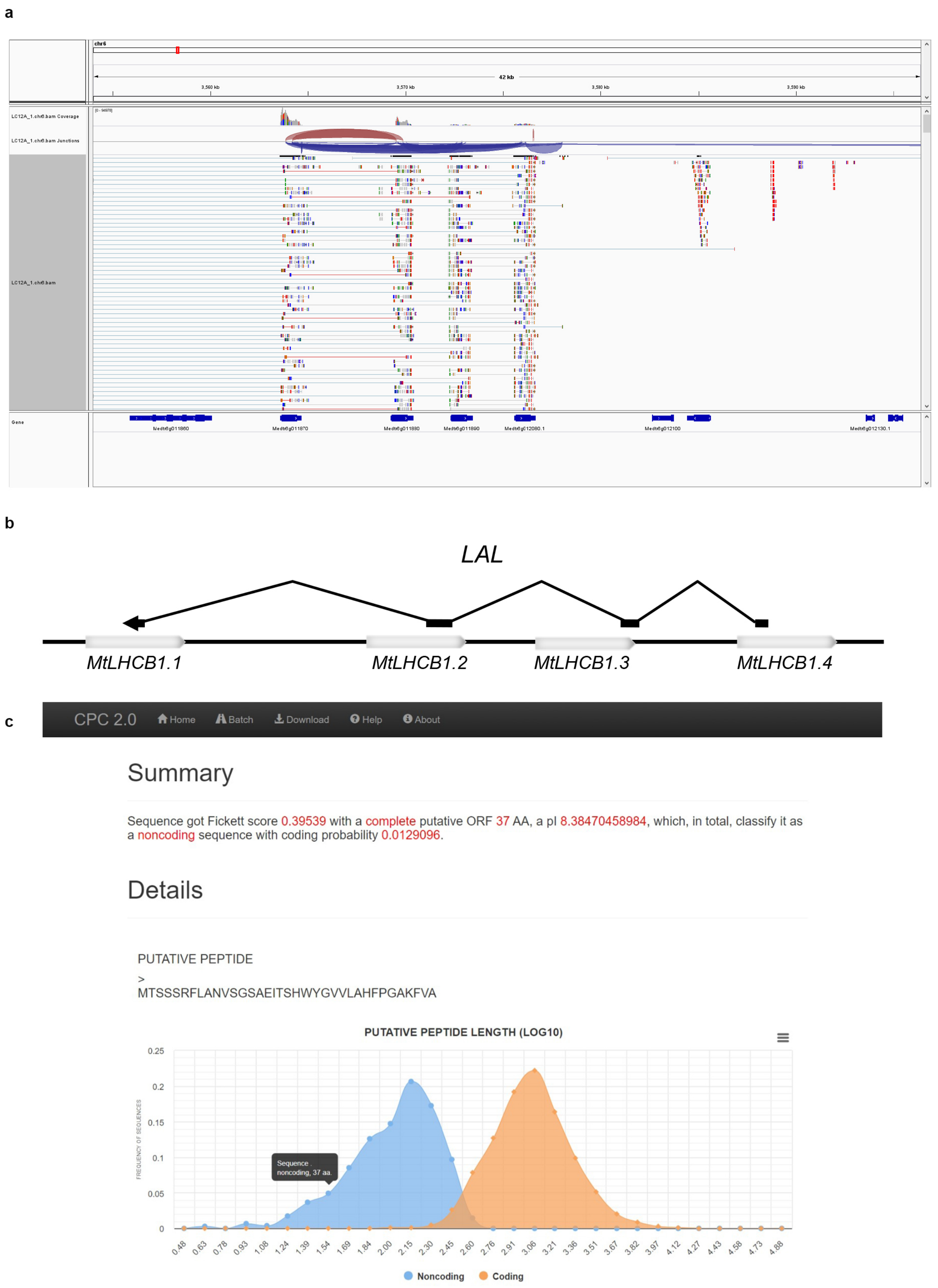

Supplementary Fig. 2 *LAL* was as an antisense transcript of *MtLHCB1s*. (a) The IGV view of *LAL* expressed as an antisense to a *MtLHCB1s* gene cluster. (b) The scheme of *LAL* and *MtLHCB1.1*, *MtLHCB1.2*, *MtLHCB1.3* and *MtLHCB1.4* genes structure. (c) The CPC summary of *LAL*.

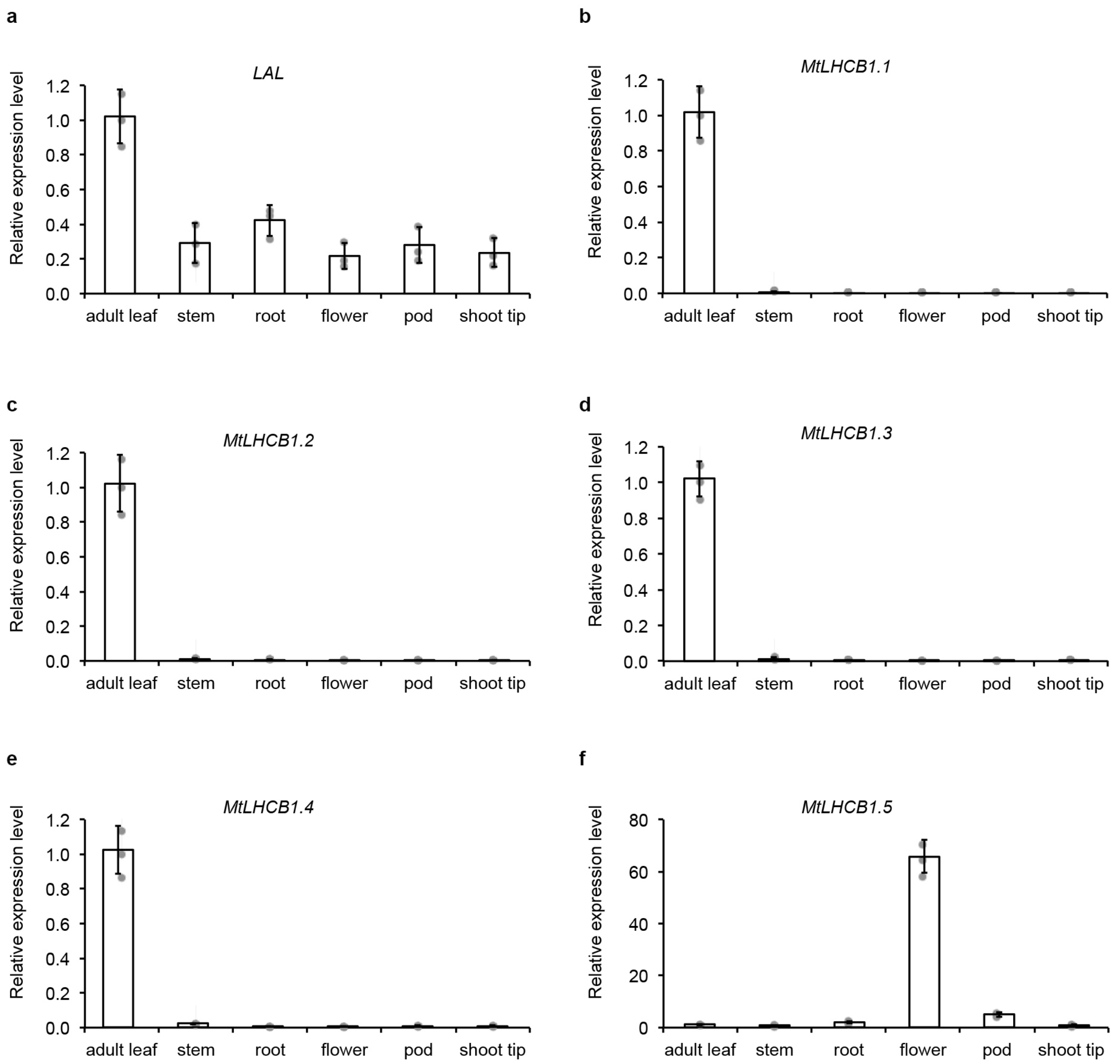

Supplementary Fig. 3 *LAL* and *MtLHCB1s*' expression in various tissues. (a) The expression level of *LAL* in adult leaf, stem, root, flower, pod and shoot tip by qRT-PCR in *M. truncatula* WT plants. (b) The expression level of *MtLHCB1.1* in adult leaf, stem, root, flower, pod and shoot tip by qRT-PCR in *M. truncatula* WT plants. (c) The expression level of *MtLHCB1.2* in adult leaf, stem, root, flower, pod and shoot tip by qRT-PCR in *M. truncatula* WT plants. (d) The expression level of *MtLHCB1.3* in adult leaf, stem, root, flower, pod and shoot tip by qRT-PCR in *M. truncatula* WT plants. (e) The expression level of *MtLHCB1.4* in adult leaf, stem, root, flower, pod and shoot tip by qRT-PCR in *M. truncatula* WT plants. (f) The expression level of *MtLHCB1.5* in adult leaf, stem, root, flower, pod and shoot tip by qRT-PCR in *M. truncatula* WT plants. *MtUBIQUITIN* was used as the internal control. The expression level in adult leaf was set at 1.0. Error bars represent the SD from three biological replicates. *n*=3.

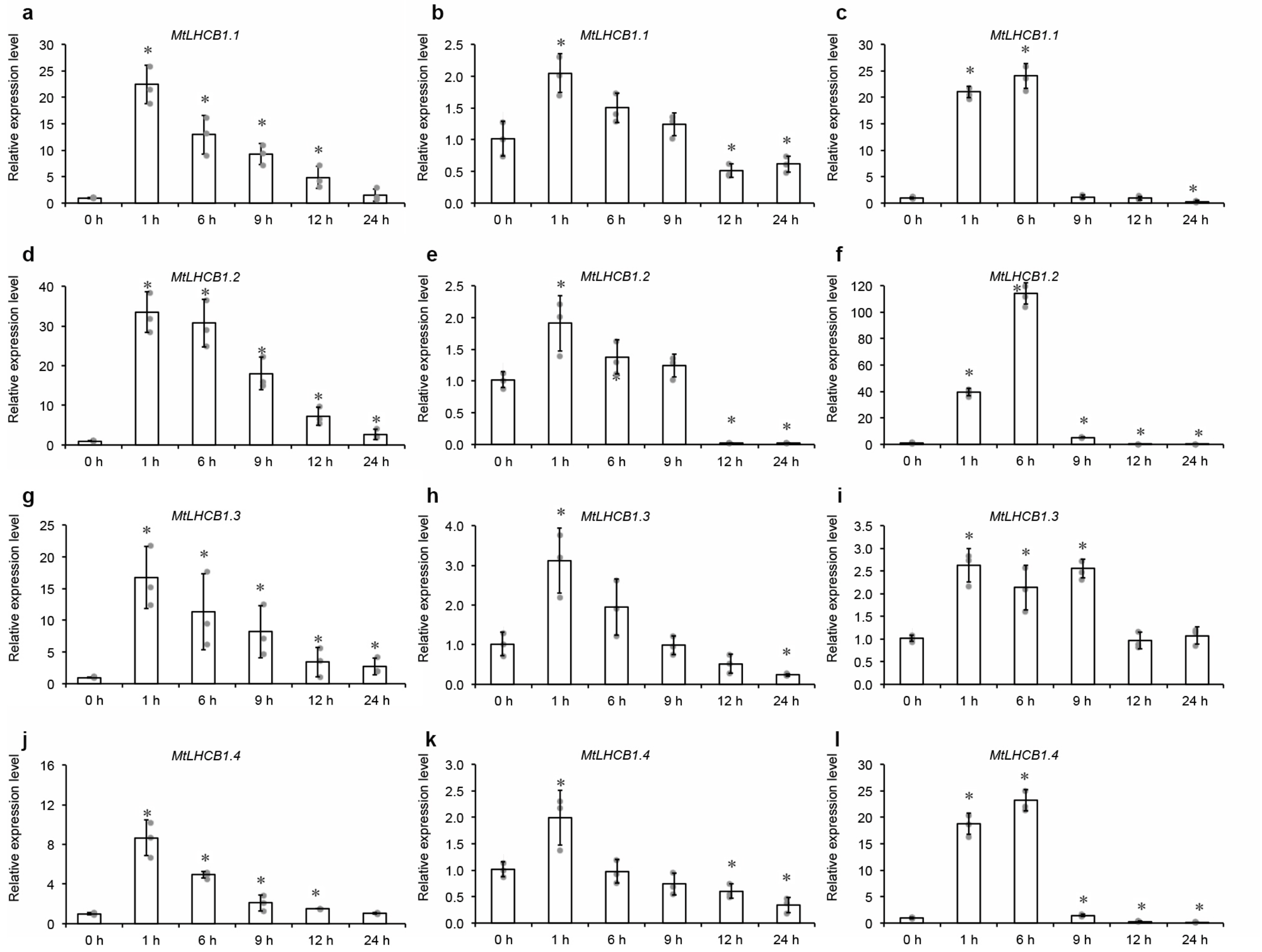

Supplementary Fig. 4 *MtLHCB1s*' expression under salinity, ABA and H_2O_2 treatment. The expression level of *MtLHCB1.1* at different time points by qRT-PCR in *M. truncatula* WT plants under salinity treatment (a), ABA treatment (b), or H_2O_2 treatment (c). The expression level of *MtLHCB1.2* at different time points by qRT-PCR in *M. truncatula* WT plants under salinity treatment (d), ABA treatment (e), or H_2O_2 treatment (f). The expression level of *MtLHCB1.3* at different time points by qRT-PCR in *M. truncatula* WT plants under salinity treatment (g), ABA treatment (h), or H_2O_2 treatment (i). The expression level of *MtLHCB1.4* at different time points by qRT-PCR in *M. truncatula* WT plants under salinity treatment (j), ABA treatment (k), or H_2O_2 treatment (l). *MtUBIQUITIN* was used as the internal control. The expression level in 0 hour was set at 1.0. Error bars represent the SD from three biological replicates. Columns labeled with asterisks indicate significant differences from those in 0 hour (*$P < 0.05$, student's t test). n=3.

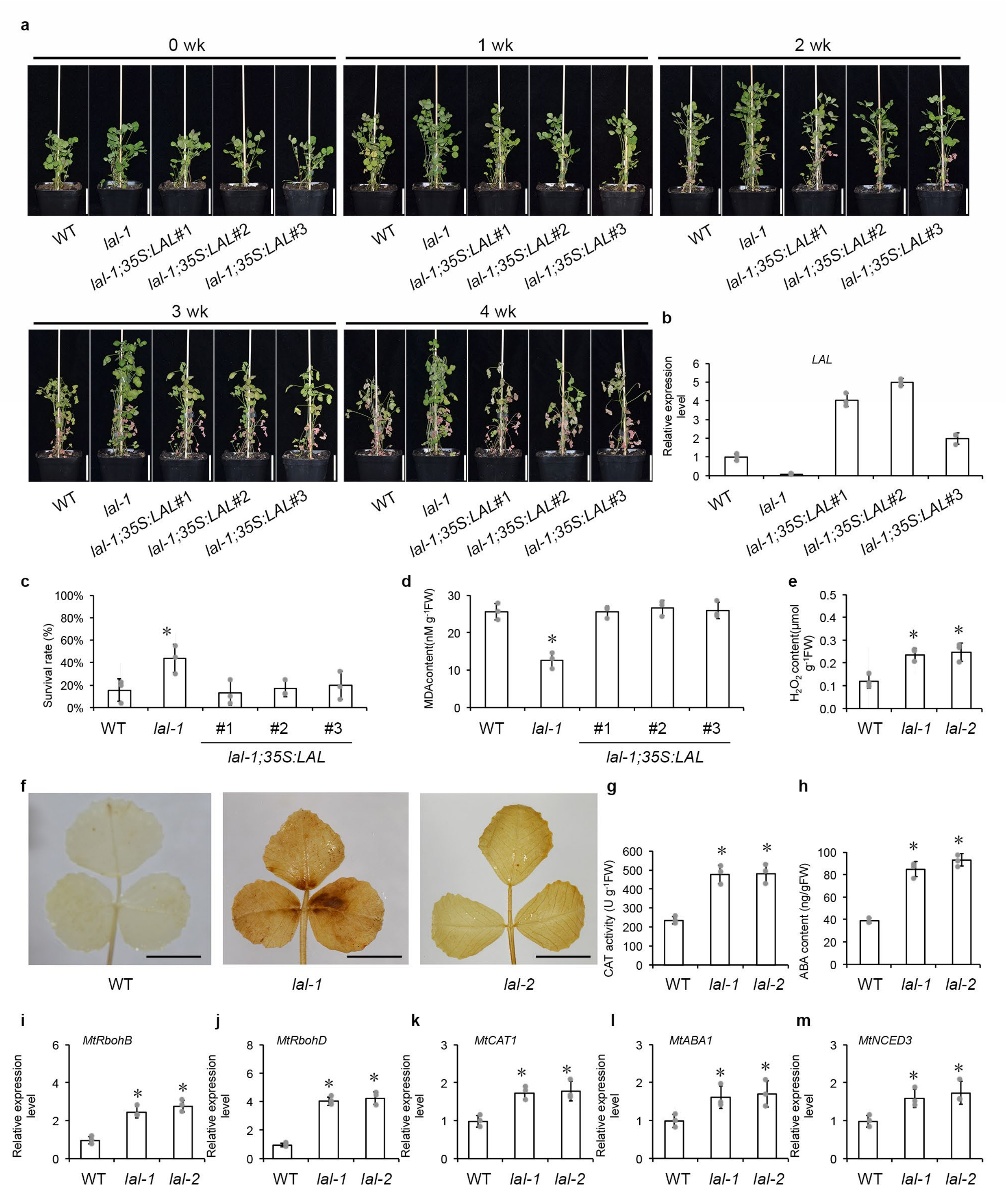

Supplementary Fig. 5 Restoration of the WT response to salinity by overexpressing *LAL* in *lal-1*. (a) Four-week-old plants of WT, *lal-1*, *lal-1;35S:LAL*#1, *lal-1;35S:LAL*#2 and *lal-1;35S:LAL*#3 after a four-week successive exposure to 50, 100, 150 and 200 mM NaCl. Bar = 5 cm. (b) The expression level of *LAL* in WT, *lal-1*, *lal-1;35S:LAL*#1, *lal-1;35S:LAL*#2 and *lal-1;35S:LAL*#3 by qRT-PCR. *MtUBIQUITIN* was used as the internal control and the expression level in WT was set at 1.0 in qRT-PCR. (c) The plant survival rates measured after the four-week treatment of salinity.

(d) The MDA content of WT, *lal-1*, *lal-1;35S:LAL*#1, *lal-1;35S:LAL*#2 and *lal-1;35S:LAL*#3. Error bars represent the SD from three biological replicates. Columns labeled with asterisks indicate significant differences from those in WT (*$P < 0.05$, student's t test). (e) The H_2O_2 contents of WT, *lal-1* and *lal-2* mutants. (f) The DAB staining of WT, *lal-1* and *lal-2* mutants. Bar = 1 cm. (g) The CAT activity in WT, *lal-1* and *lal-2*. (h) The ABA contents of WT, *lal-1* and *lal-2* mutants. (i) The expression level of *MtRbohB* in WT, *lal-1* and *lal-2* by qRT-PCR. (j) The expression level of *MtRbohD* in WT, *lal-1* and *lal-2* by qRT-PCR. (k) The expression level of *MtCAT1* in WT, *lal-1* and *lal-2* by qRT-PCR. (l) The expression level of *MtABA1* in WT, *lal-1* and *lal-2* by qRT-PCR. (m) The expression level of *MtNCED3* in WT, *lal-1* and *lal-2* by qRT-PCR. n=3.

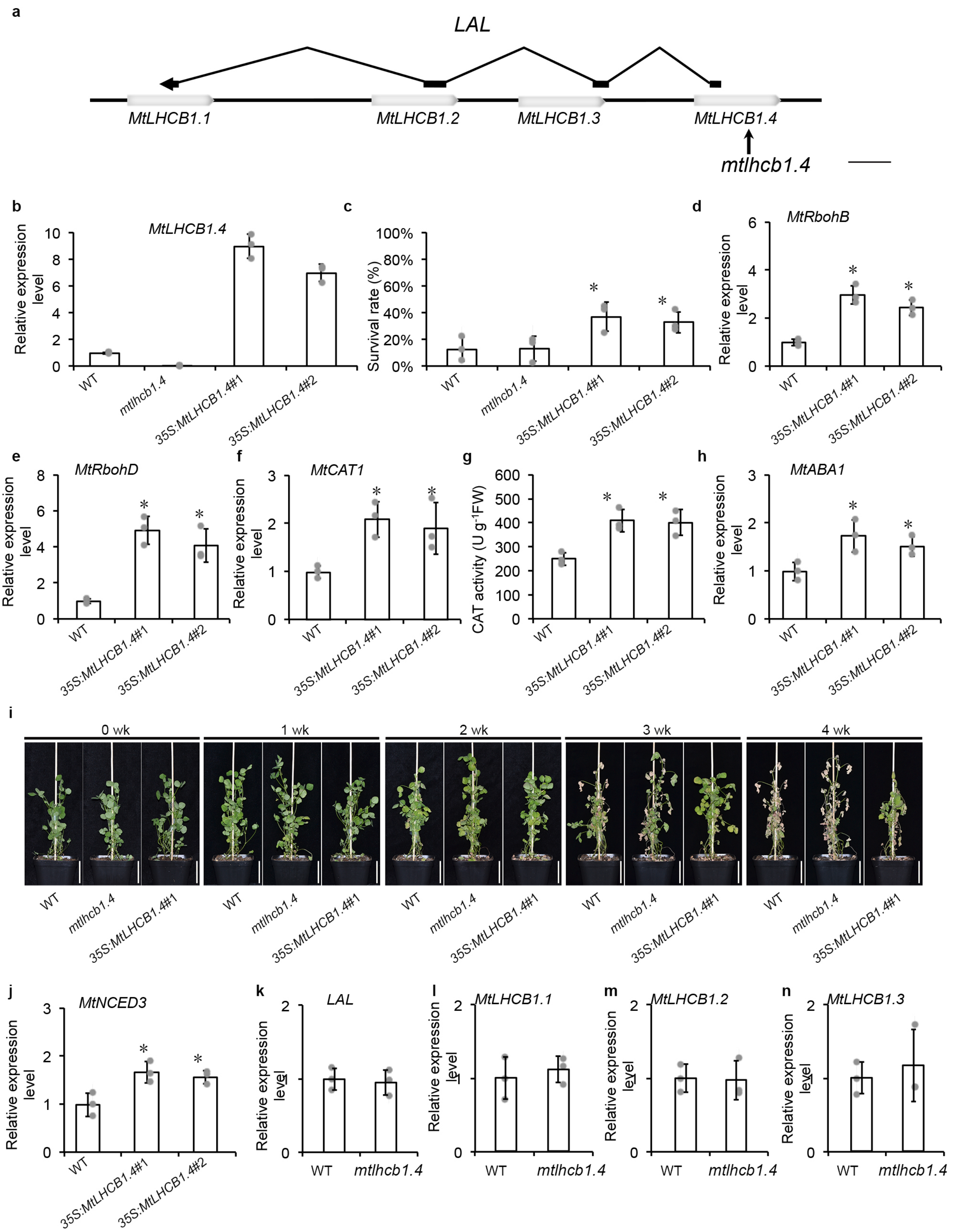

Supplementary Fig. 6 Overexpressing *MtLHCB1.4* enhanced salinity tolerance. (a) The scheme of *LAL* and *MtLHCB1.1*, *MtLHCB1.2*, *MtLHCB1.3* and *MtLHCB1.4* genes structure and *Tnt1* insertion in *MtLHCB1.4* of *mtlhcb1.4*. Empty boxes represent *MtLHCB1*s, and black boxes represent *LAL*. One arrow indicates the position where *Tnt1* inserts in *MtLHCB1.4*. (b) The expression level of *MtLHCB1.4* in WT, *mtlhcb1.4*, *35S:MtLHCB1.4*#1, and *35S:MtLHCB1.4*#2

plants by qRT-PCR. (c) The plant survival rates measured after the four-week treatment of salinity. The expression level of *MtRbohB* (d), *MtRbohD* (e) and *MtCAT1* (f) in WT, *35S:MtLHCB1.4*#1 and *35S:MtLHCB1.4*#2 by qRT-PCR. (g) The CAT activity in WT, *35S:MtLHCB1.4*#1 and *35S:MtLHCB1.4*#2. The expression level of *MtABA1* (h) in WT, *35S:MtLHCB1.4*#1 and *35S:MtLHCB1.4*#2 by qRT-PCR. (i) Four-week-old plants of WT, *mtlhcb1.4* and *35S:MtLHCB1.4*#1 plants after a four-week successive exposure to 50, 100, 150 and 200 mM NaCl. Bar = 5 cm. The expression level of *MtNCED3* (j) in WT, *35S:MtLHCB1.4*#1 and *35S:MtLHCB1.4*#2 by qRT-PCR. The expression level of *LAL* (k), *MtLHCB1.1* (l), *MtLHCB1.2* (m) and *MtLHCB1.3* (n) in *mtlhcb1.4*. *MtUBIQUITIN* was used as the internal control and the expression level in WT was set at 1.0 in qRT-PCR. Error bars represent the SD from three biological replicates. Columns labeled with asterisks indicate significant differences from those in WT (*$P < 0.05$, student's t test). n=3.

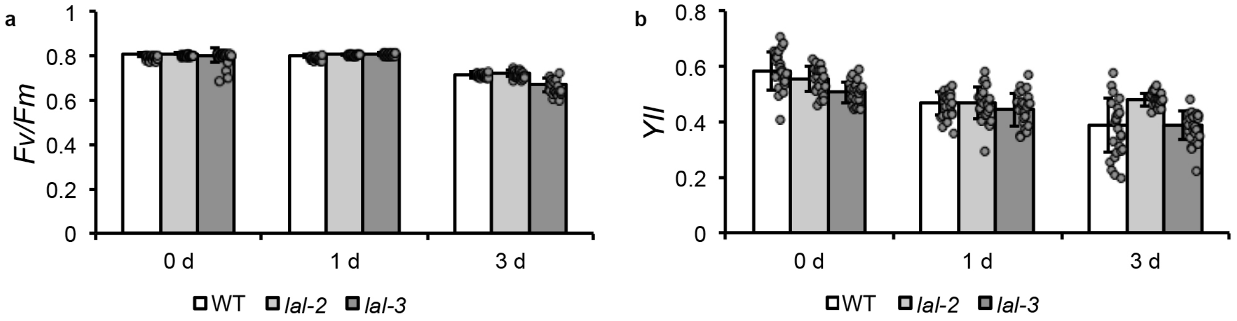

Supplementary Fig. 7 Response to salinity stress on photosynthetic parameters *Fv/Fm* and *YII* of the WT, *lal-2* and *lal-3* mutants. (a) The *Fv/Fm* of the WT, *lal-2* and *lal-3* mutants at different time points of NaCl-treatment. (b) The *YII* of the WT, *lal-2* and *lal-3* mutants at different time points of NaCl-treatment. Values are shown by mean ± SD (grey dots with black circles). *n*=25.

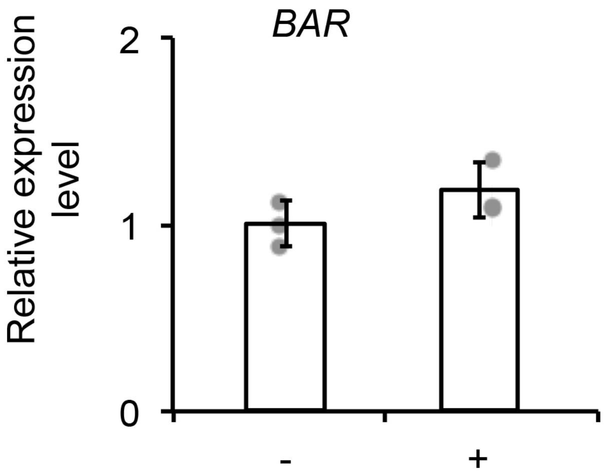

Supplementary Fig. 8 The expression of *BAR* in tobacco leaves transformed with *35S:MtLHCB1.4-GFP* with *LAL* indicated by '+' and without *LAL* indicated by '-' in *N. benthamiana*. *MtUBIQUITIN* was used as the internal control. The expression level in *35S:MtLHCB1.4-GFP* without *35S:LAL* was set at 1.0. Error bars represent the SD from three biological replicates. *n*=3.

Supplementary Table 1. DEGs at three hours and 12 hours after NaCl treatment.

Supplementary Table 2. PCR primer sequences used in this study.

Name	Forward primer	Reverse primer
MtLHCB1.1	CCTCTCGGTGAGGTGGTTGA	CCTTTCCAGTCACAATGGCT
MtLHCB1.2	AGTTATCTTAATGGGCGCCGT	GCGAGGTTCTCAATAGGTCCC
LAL	GGTGTAGTTTTGGCTCAC	TCTTCGTTCAGGCCATTGTCA
Tnt1-ReverseF	TGTAGCACCGAGATACGGTAATTAACAAGA	
Tnt1-ReverseR	CCGTATCTCGGTGCTACATTATT	
MtLHCB1.4	CATTGGGCCTCGCTGACGAC	CGGGGTCAGCGATATGGTCG
geno-lal-1-1.4	AGCATCATCAATAACCATCCTCTTC	CACGAAATTCGCACTCTTGT
MtLHCB1.5	GTGCAATGCTCGAAACCAATTGA	CATTGTTGAAGCAGCCATTGTAG
MtLHCB1.6	TACACACCCATATTCACCTTACC	AATTCTTGGCTTGAAGGGGTTAG
RiMtLHCB1.4	TGGGCTGTCCAAGTTATCTT	GTTACAATGGCCTGAACGAA
geno-lal-2-3	AGCTATTGTAACCGGAAAAGGAC	TCATGGGTTACTGGTGATCCT
MtLHCB1.3	AGCTATTGTAACCGGAAAAGGAC	TGGTGTAGTTTTGGCTCACTTT
c-LAL	ACTGTGTATGACCTCGAGTTCAC	GTAACCCAAGTCTAGTCCATGCA
c-MtLHCB1.4	ATGGCTACATCAACAATGGCTCT	CTTTCCGGGGACAAAGTTTGT

5'RACELAL	GCCAAAACTACACCATACCAATG	
3'RACELAL	AGAAATCACTTCTCATTGGTAT	
5'RACEMtLHCB1.4	CTTTCCGGGGACAAAGTTTGT	
TransientAssayqRTMtLHCB1.4	TTTGCTTTTAACTAAGTTGC	GTGTATGACCTCGAGTTCAC
MtCAT1	CTGGAGTTCAGACACCCGTC	AAGGTTTCAGGGCTACCACG
MtRbohB	GCTCGCTCTGCTCTTATTGC	TGCGCTTGTAGACACTACGC
MtRbohD	ACATGGCTCAGGAGCAAGAC	TGAAGAAGGCGTGGAAAGTC
MtABA1	GGCTTCCACCTACAAGGCAT	TCCTCCAACTCTTCCCGGAT
MtNCED3	CTACCCAAAACCTCCGACCC	ATTGGGTTACCGGGTGTTCC
siRNALAL	GCGACCTCGAGTTCACGGTTCTTGGC	
BAR	ACACCCACCTGCTGAAGTCCCTG	CCAGAAACCCACGTCATGCCAGT
MtUBIQUITIN	CTGACAGCCCACTGAATTGTGA	TTTTGGCATTGCTGCAAGC
35SF	GCACAATCCCACTATCCTTC	
MtU6	GAGAAGATTAGCATGGCCCCT	
L25	AATCTGACCCCAAGGCACAG	ACGAGGGTACTTGGGGTTTC
