# Supplementary material for: The long noncoding RNA LAL contributes to salinity tolerance by modulating LHCB1s’ expression in Medicago truncatula
Source: Commun Biol. 2024 Mar 8;7:289. doi: 10.1038/s42003-024-05953-9 (PMC10923924; doi:10.1038/s42003-024-05953-9)
Supplement: Supplementary file 2 — Description of Additional Supplementary Files [file 42003_2024_5953_MOESM2_ESM.pdf]

Description of Additional Supplementary Files

File name: Supplementary Data 1

Description: DEGs at three hours and 12 hours after NaCl treatment.

File name: Supplementary Data 2

Description: Numerical source data for Figures.
